# Contemporary Review of Outcomes in Patients Undergoing Transcatheter Aortic Valve Replacement with Concomitant Mitral Stenosis

**DOI:** 10.2174/011573403X385102251003105530

**Published:** 2025-10-20

**Authors:** Asra Butt, Gift Echefu, Derek Geeslin, Latifah Ahmad, Daniel Craig, Olisaemeka Ndubisi, Mannu Nayyar, Uzoma N. Ibebuogu

**Affiliations:** 1 Division of Cardiovascular Diseases, College of Medicine, University of Tennessee Health Sciences Center, Memphis, TN 38163, United States of America;; 2 Department of Internal Medicine, College of Medicine, University of Tennessee Health Sciences Center, Memphis, TN 38163, United States of America;; 3 Department of Internal Medicine, University of Galway, Galway, Ireland

**Keywords:** Transcatheter aortic valve replacement, mitral valve stenosis, cardiovascular mortality, trans-mitral mean gradient, mitral valve area, multivalvular cardiac disease

## Abstract

**Introduction:**

Multivalvular cardiac disease has been associated with increased mortality and morbidity as compared to isolated valve disease. The treatment of choice for combined degenerative aortic and mitral stenotic disease is considered to be double valve surgery, but it is associated with poorer outcomes when compared to isolated valve surgical correction, especially in high-risk populations. There is considerable interest in utilizing transcatheter therapies in multivalvular disease. The prevalence and outcomes of patients undergoing transcatheter aortic valve replacement (TAVR) for aortic stenosis (AS) with concomitant mitral stenosis (MS) have not been studied extensively. Currently, there are no specific recommendations or guidelines for managing these patients.

**Methods:**

A search of PubMed and Cochrane databases was performed for clinical studies of patients undergoing TAVR for severe AS with concomitant MS.

**Results:**

Our research demonstrates that most patients undergoing TAVR for severe AS with concomitant mitral valve stenosis tend to be elderly with degenerative valve disease. Short-term outcomes, such as postoperative hospital stay, as well as long-term outcomes, such as 1-year mortality, are worse in TAVR with severe MS. TAVR in mild to moderate MS shows no significant differences in outcomes compared with TAVR for isolated severe AS.

**Discussion:**

Most studies have shown that in subjects undergoing TAVR with concomitant MS, severe MS is associated with worse outcomes, while mild to moderate MS shows no significant difference compared with the absence of MS. Limitations of our study are mainly related to the small number of high-quality clinical trials examining TAVR in patients with combined AS and MS.

**Conclusion:**

With the aging population, TAVR has been a more attractive option for the treatment of severe symptomatic AS. The optimal treatment of patients with AS along with MS is not clear. Thus, further research is needed in this field.

## 1 INTRODUCTION

The incidence of cardiac valvular disease has risen alongside the increase in average life expectancy. In the developed world, aortic valve stenosis resulting from degenerative and aging processes is now the predominant form of valvular disease [1-6]. Currently, transcatheter aortic valve replacement (TAVR) is being adopted as a guideline-recommended intervention for severe aortic valve stenosis (AS) in patients who are intermediate to high-risk for surgical aortic valve replacement (SAVR) [7-10]. It has been estimated that about 290,000 patients with severe AS in North America and Europe are candidates for TAVR, and about 27,000 patients become candidates for TAVR annually [10-15].

The 5-year outcomes following TAVR for severe symptomatic aortic stenosis have been reported as noninferior to SAVR. TAVR is comparatively less invasive, and with advancements in medical devices, has shown shorter hospital stays as well as a a reduction in 30-day complication rates compared to SAVR [16-19]. Thus, an upward trend has been observed in the number of TAVR being performed worldwide [15]. Despite the continuous improvement in TAVR outcomes over time, certain factors persistently correlate with less favorable results. One such factor is the coexistence of multivalvular disease, especially mitral valve disease, which has been shown to portend poorer outcomes [20]. The hemodynamic impairment associated with multiple valve disease may pose a diagnostic challenge with echocardiography in accurately assessing the severity of each diseased valve. Thus, late presentation and resultant increase in morbidity further elevate their surgical risk [21, 22]. Cases of concurrent surgical procedures aimed at addressing multiple dysfunctional valves within a single operation have been documented; however, this approach is associated with a threefold increase in surgical risk compared to isolated-valve procedures, even with comparable preoperative profiles [22-24]. In the current era of transcatheter interventions, most research on multivalvular heart disease has focused on concurrent mitral regurgitation and TAVR, suggesting that co-existing mitral regurgitation is an independent risk factor associated with poor outcomes and prognosis following TAVR [9]. However, evidence regarding the incidence and outcomes of TAVR with concurrent mitral stenosis (MS) remains limited at present.

Mitral stenosis (MS) has been reported in approximately 10-12% of patients with severe AS, with an even higher incidence in elderly patients [25-29]. All forms of mitral disease, including mitral annular calcification (MAC), have been shown to be linked to poor outcomes in subjects undergoing TAVR [29-40].

The aortic and mitral valves share a common fibrous septum, and the coordination of their function is essential to ensure forward flow [41]. The proposed pathogenesis of MS occurring in the background of AS has been ascribed to elevated left ventricular (LV) pressures and injury to the mitral valve due to this proximity. Thus, TAVR can affect the function of the mitral valve by changing the morphology of the left ventricular outflow tract (LVOT) and the shape of the mitral valve annulus [42, 43].

The 2020 guidelines from the American Heart Association and American College of Cardiology (ACC) recommend concomitant mitral valve surgery in patients with moderate to severe mitral stenosis who are undergoing cardiac surgery for other indications [44]. There are no current recommendations to direct the timing and specific management of moderate to severe mitral stenosis in patients undergoing TAVR in the transcatheter valve era. Concomitant MS may influence clinical decision-making when choosing between SAVR and TAVR in patients at intermediate to high surgical risk. Consequently, some patients who are poor candidates for dual valve surgery may be considered for TAVR alone [45, 46]. This review article summarizes and reflects on the results of studies investigating the prevalence and clinical implications of MS in patients undergoing TAVR for aortic stenosis.

## 2 METHODS

Two of the authors (AB and LA) conducted a systematic literature search across various databases, including Cochrane Central Register of Controlled Trials, PubMed (MEDLINE), Google Scholar, Cumulative Index to Nursing and Allied Health Literature (CINAHL), and EMBASE databases, to find relevant research studies published up to May 2024. The search keyword included the terms “TAVI”, “TAVR”, “Transcatheter aortic valve Replacement”, “Transcatheter aortic valve Implantation”, and “Mitral Valve stenosis”. A review of the list of references from the studies was manually performed to identify further relevant articles.

The eligibility criteria for this study were trials of adult patients (18 years and older) who had undergone TAVR with all degrees of mitral valve stenosis. The analysis focused on the outcomes reported at 1 month and at least one year after TAVR. Case reports, animal trials, abstracts, and studies not reporting the specified clinical outcomes were excluded. Only observational cohort studies and randomized controlled trials (RCTs) were chosen to be included in the review (see Fig. **1**).

Two reviewers (AB and LA) independently reviewed the abstracts and titles for articles that fulfilled the previously stated criteria. All texts of possibly appropriate studies were further evaluated by the two reviewers to confirm inclusion. Discrepancies among the reviewers were addressed by the third reviewer (GE).

## 3 RESULTS

### 3.1 Prevalence of Mitral Stenosis Among TAVR Patients

MS has two main etiologies, namely rheumatic and degenerative diseases. In rheumatic disease, the mitral valve is usually the first to be affected by inflammation, leading to fusion of the commissures and resultant stenosis. Degenerative MS, the most common etiology in aging populations in industrialized countries, is caused by progressive calcification, and there is no fusion of the commissures; therefore, it results in a lesser degree of stenosis [47]. The prevalence of all types of MS in patients undergoing TAVR has been estimated to range from 7% to 20% depending on the study [29, 48-51]. The proportion of degenerative *versus* rheumatic MS in TAVR is not as well known, as many trials examining TAVR and concomitant MS do not specify the type of MS. In a study by Asami *et al*., 1339 patients underwent TAVR between August 2007 and December 2015, of which 18.1% of TAVR patients had mitral stenosis; 62.5% were considered degenerative, and 37.5% were rheumatic [48]. In the registry paper on the transcatheter valve therapies by Joseph *et al*, most patients likely had degenerative disease, given the mean age of the cohort was 82 years. This is supported by the fact that only 1.4% of patients in this registry underwent mitral valve intervention in the year following TAVR [29]. Similarly, most of the publications that have studied TAVR and MS include subjects with advanced ages, suggesting a higher incidence of degenerative MS compared to rheumatic MS [52].

### 3.2 Outcomes of TAVR in Patients with Concomitant Mitral Stenosis

Different studies have employed various modalities to diagnose MS and used varying criteria to grade the severity of valve stenosis as mild, moderate, or severe. Some authors used the mitral valve area, while others graded MS by valve gradient. A considerable number of patients have coexisting mitral annular calcification and/or mitral regurgitation, which adds further complexity in accurately determining the degree of mitral stenosis. Short and long-term outcomes reported by studies (Table **1**) have included 30-day cardiovascular (CV) mortality, hospitalization for heart failure, in-hospital mortality rates, and stroke [53, 54].

The retrospective study of 44,755 patients undergoing TAVR from November 2011, to September 2015, enrolled in the Society of Thoracic Surgeons /American College of Cardiology Transcatheter Valve (STST/ACC) Registry, investigated the in-hospital and one-year follow-up results for 5,201 patients with co-existing severe aortic stenosis and MS [29]. In this study, the definition of non-severe MS was a mitral valve area between 1.51- 4.0 cm^2^ (3,987 patients), and severe MS as 1.5 cm^2^ or less (1,214 patients). The primary outcome was specified as the composite of all-cause mortality, cerebrovascular accident (CVA), hospital admission for heart failure, and intervention for mitral valve disease at 12 months post TAVR. After adjusting for baseline characteristics (dialysis dependence, functional class IV New York Heart Association heart failure, and multiple valvular insufficiencies), the occurrence of MS did not significantly increase the risk of short-term outcomes of death or a composite of in-hospital death and CVA among patients undergoing TAVR. However, at the 1-year follow-up, patients with severe MS had a significantly increased risk for the composite primary outcome, 1-year mortality (*p*= 0.039), heart failure-related hospitalization (*p*=0.012), and need for subsequent mitral valve intervention compared to the no MS group. The elevated baseline risk profile of the individuals with severe MS following TAVR may elucidate the heightened incidence of 1-year adverse events. Limitations of the study included the obscurity regarding the methods for assessing MV area and the etiology of MS, whether through echocardiography or cardiac catheterization, as well as follow-up information limited to one year, with no data regarding functional status and exercise capacity [29].

Similar results were reported by another study comparing 1-month and 12-month outcomes in 176 patients with concomitant MS in the Swiss Transcatheter Aortic Valve Implantation registry (STAVI) [48]. The degree of MS was defined by MVA; normal >2.5 cm^2^, mild 1.5–2.5 cm^2^, moderate 1.0–1.5 cm^2^, and severe <1.0 cm^2^) and mean mitral valve gradient; mild <5 mm Hg, moderate 5–10 mm Hg, and severe >10 mm Hg. The primary endpoint of the study was cardiovascular death 1 year after TAVR. Secondary endpoints included all-cause mortality, disabling stroke, and myocardial infarction at 30 days and at 1 year. The advantage of this study over the STS/ACC registry analysis is that the authors had access to raw echocardiographic data, which was interpreted by independent reviewers to minimize subjectivity in the assessment of disease severity. MS was noted in 18.1% of subjects; 110 (62.1%) had degenerative and 67 (37.9%) had rheumatic MS. Mild MS was present in 148 (15.2%) subjects, while moderate or severe MS was noted in 28 patients (2.9%). The 1-year CV death risk was 21.4% (no MS) *vs* 8.7% ( MS) with an HR of 3.64 and a 95% confidence interval (CI) of 2.38–5.56. The incidence of disabling stroke at 1 year was 7.1% ( MS) *vs.* 3.0% ( no MS) with adjusted HR 2.98, 95% confidence interval CI 1.46–6.09. Even though MS was mild in the majority of cases and did not result in secondary pulmonary hypertension or manifest valvular atrial fibrillation, it was still associated with a significantly increased risk of cardiovascular death and disabling stroke at 30 days and 1 year. The authors also noted that the difference in CV death and disabling stroke in MS *vs* non-MS groups emerged within 30 days of the procedure and was mainly driven by the outcomes of rheumatic MS. Subjects with rheumatic MS had a 4.8-fold increased risk of cardiovascular death and a 4.2-fold increased risk of disabling stroke at 30 days post-TAVR. Subjects with degenerative MS had a 4.0- and 3.9 times increased risk of cardiovascular death or disabling stroke 1 year post TAVR. The authors hypothesized that the higher risk of rheumatic MS compared to degenerative MS could be related to a higher proportion of advanced stages of MS in the cohort with rheumatic MS [48].

In the study by Sannino *et al*, the severity of MS was determined by echo using MV mean gradient (MMG) and by valve area. Patients were stratified into MMG <5 mm Hg, MMG ≥5 to <10 mm Hg, MMG ≥10 mm Hg, and MV area >1.5 cm^2^ and MV area ≤ 1.5 cm^2^. The all-cause mortality at 5 years was noted to be higher in subjects with MMG ≥ 10 mm Hg, with a HR of 2.91 (*p*= 0.02). However, no significant differences were observed in all-cause mortality at 1 month and 12 months in the MMG groups. No difference was noted in the 5-year mortality rate when stratified by MVA [49].

A multinational study identified 157 patients who underwent TAVR between 2007 and 2017 with concomitant moderate or severe MS, defined with baseline echocardiography as a mean transmitral gradient ≥ 5 mm Hg without severe mitral valve regurgitation [50]. Patients were grouped into the MS and no MS groups. The 1-month mortality rate between the MS group and the non-MS group did not show any significant difference (3.8 *vs.* 5.5% respectively). Similarly, after a follow-up of 3 years, there were also no differences between the groups in mortality (MS: 35.0% *vs* no MS: 36.2%), heart failure rehospitalization (MS: 21%, no MS: 21.7%), or cardiovascular mortality (MS: 21.0%, no MS: 22.4%). They also noted that patients with MS with MMG ≥10 mm Hg had similar short and late outcomes in comparison to the rest of the study cohort, and failed to show any relationship between outcomes and severity of MS. MS patients compared to non-MS patients had similar improvements in their functional status and exercising capacity [50].

In addition to MS, mitral annular calcification (MAC) has been described as a predictor of elevated all-cause mortality, conduction system complications, and cardiovascular mortality following TAVR. Schaefer *et al*. [51] reported on the outcomes of 1,058 subjects with MAC, mild MS, moderate MS, or severe MS who underwent TAVR between 2009 and 2017, at both 30 days and an average follow-up of 703.5 days. The authors defined the severity of MS based on the mean pressure gradient across the MV, with a mean pressure gradient of 2 to 5 mm Hg classified as mild MS, 5 to 10 mmHg as moderate MS, and 10 to 15 mm Hg as severe MS. The 30-day all-cause mortality was reported at 7.5% among all patients, with the highest rates in moderate and severe MS. The 30-day all-cause mortality was 3.4% for MAC, 5.9% in mild MS, 15.0% in moderate MS, and 72.7% in severe MS. The *p*-value was < 0.001. No significant differences were observed between the cohorts for stroke, myocardial infarction, major bleeding, and permanent pacemaker implantation. The Kaplan–Meier survival analysis at 703.5 days showed the lowest long-term survival in severe MS, while there were no significant differences in patients with MAC, mild MS, or moderate MS [51]. The results of this study warrant further investigation, as the severe MS population was too low to detect an impact. Furthermore, a high use of the transapical approach was observed, which is known to contribute poorly to post-TAVR outcomes [53], and MAC severity was not objectively quantified.

Similarly, a prospective study by Mesnier *et al* evaluated the effect of MAC and MS in 1177 patients who had undergone TAVR. MS was defined as MMG ≥5 mm Hg, with no severe MR. The authors reported MAC-MS but not MAC no-MS as an independent predictor for all-cause mortality at 1 year post-TAVR. All-cause death at 30 days and 1 year was higher in the MAC-MS group compared to the no MAC group [55].

In the post-TAVR outcomes study by Okai *et al*. [56], the primary endpoint was defined as the incidence of acute decompensated heart failure (ADHF) within 72 hours post-TAVR in the 156 study participants. Among these, 15 were found to have MS, defined as a mean mitral valve pressure gradient of ≧5 mm Hg. In the study, the authors defined ADHF as worsening of HF symptoms, including dyspnea, along with signs of pulmonary edema or impaired perfusion, leading to the use of intravenous diuretics, vasodilator therapy, inotropic support, or the need for respiratory support. Multivariable logistic regression analysis showed that MS, along with a decreased mean aortic valve gradient (mAVPG), was associated with ADHF. Although this study did not measure the outcomes of these patients based on the severity of MS, the presence of MS was observed to be the strongest risk factor for patients developing ADHF. The 2-year mortality rate was noted to be 34.5% for the MS cohort, as compared to 11.6% (95% CI, 7.1% to 18.6%) for the non-MS cohort. The hemodynamic alterations caused by variable anesthesiologic regimens during procedures could have contributed to the heart failure outcomes. Additionally, the MS group (n = 15) in the study was not powered to detect a significant impact [56].

Kato *et al* [57] retrospectively studied subjects who had undergone TAVR or SAVR for severe symptomatic aortic stenosis. They identified 184 patients undergoing AVR (55 TAVR, 129 SAVR) and matched them with 362 controls with severe aortic stenosis, without MS, who underwent AVR (TAVR or SAVR). The study defined MS as a mean transmitral gradient ≥4 mm Hg. The patients were stratified as MVA above 2.0 cm^2^ or ≤2.0 cm^2^. The mean MVA was noted to be increased after AVR by 0.26±0.59 cm^2^ (*P*<0.01). In the 107 subjects with MS before AVR, MVA increased to >2.0 cm^2^ after AVR in 49%. Kato *et al* described this group of patients with increasing MVA after AVR as having “pseudo-MS or mild MS”. Pseudo-MS in this context means that the stenosis of the MV is not due to the structure of the mitral valve itself, but due to limited mitral leaflet opening related to the stenotic aortic valve and associated LVOT changes. In these pseudo-MS patients, MVA stenosis improved after isolated AVR. In 51% patients, the MVA remained ≤2.0 cm^2^, indicating stenosis caused by structural changes in the mitral valve itself (“true MS”). True MS, pseudo-MS, and controls had similar perioperative outcomes. However, after follow-up of median 2.9 (0.7–4.9), true MS was shown to be an independent predictor of all-cause mortality with an adjusted hazard ratio of 1.88; 95% CI, 1.20–2.94; *P*<0.01. However, pseudo-MS was not shown to be an independent predictor of mortality, with an adjusted HR of 1.38 (95% CI, 0.99–1.91; *p*= 0.053) [57].

Al-Khadra *et al*. [58] published a retrospective analysis of subjects undergoing TAVR from the US National Inpatient Sample database from 2011 to 2014, using ICD-9-CM codes to identify subjects who had undergone TAVR. About 1.43% of TAVR patients had concurrent MS. The TAVR patients with concurrent MS had an increased in-hospital mortality of 5.1% and an incidence of major adverse cardiac events of 9.0%, as compared to non-MS TAVR patients with 3.5% and 7.1%, respectively [58].

Similar to the above analysis, Kurpad *et al*. [59] reported short-term outcomes of patients post-TAVR from the US National Inpatient Sample database from 2015 to 2020. TAVR patients with MS, compared to subjects with no MS, had a greater incidence of complete heart block, acute respiratory failure, permanent pacemaker placement, and a longer duration of hospital stay of 5.13 *versus* 3.85 days. There was a greater in-hospital mortality in the MS group, but it was not statistically significant [59].

In a metanalysis observing effect of multivalvular cardiac disease on TAVR outcomes by Siddiqi *et al* [60], all grades of MS (from mild-severe) compared to no MS had no significant impact on the 1 month or 1-year mortality (HR for 1 month mortality was 1.60 with a 95% CI: 0.71-3.63 and a *p* value of 0.26; HR, 1 year mortality was 1.90 with a 95% CI: 0.98-3.69 and a p-value of 0.06). It was noted that the mortality at the end of 1 year was greater in subjects with severe MS when compared to those without MS (HR: 1.67; 95% CI: 1.03-2.70; *p*= 0.04) [60].

Ali *et al*. [53] used the U.S. Nationwide Readmission Database to assess the outcomes of 217,147 patients who underwent TAVR from 2016 to 2019, using ICD-10-CM codes. A total of 2140 (0.98%) had concurrent MS. The primary outcome, the overall 30-day all-cause readmission rate for all TAVRs, was 12.4%. TAVR patients with MS had a higher rate of 30-day readmission compared to non-MS patients (15.8% *vs* 12.3%, aOR 1.22, CI: 1.03-1.45, *p*< 0.01). Additionally, the TAVR with MS cohort compared to the non-MS cohort had a lengthier hospital stay (5.7 *vs.* 4.3 days) and higher hospitalization costs ($55,157 *vs.* $50,239). The in-hospital mortality was noted to be higher in the MS group compared to the non-MS group (2.1% *vs.* 1.5%); however, this was not a significant difference [53].

### 3.3 Outcomes of TAVR in Patients with Rheumatic Mitral Valve Stenosis

Overall, there is limited experience reported in the literature when it comes to outcomes and incidence of TAVR with concomitant MS in patients with RHD. It is rare to have isolated aortic valve disease in rheumatic heart disease (RHD), with many patients having concurrent mitral valve disease. Most patients with RHD end up requiring only mitral valve surgery in isolation. TAVR for aortic valve disease is mostly performed in these patients after a previous mitral valve intervention [61, 62]. A study by Russell *et al*. described a database from Australia of 17,000 heart valve surgeries that included 1,384 cases of RHD. It was noted that subjects undergoing surgery for RHD were more likely to be female, of a younger age, and of indigenous ethnicity, as compared to non-RHD patients. Additionally, it was reported that 23% of patients underwent isolated aortic valve operations, while 20% underwent combined aortic and mitral valve surgeries [63].

In the study by Asami *et al* [48], there were 176 patients who underwent TAVR with concomitant MS. Out of these, 66 patients had MS secondary to RHD. They found that subjects with rheumatic MS, when compared to those without MS, have a poorer short-term outcome with a 4.8 times higher risk of death from cardiovascular causes and a 4.2 times higher risk of disabling stroke at 1 month. Similarly, rheumatic MS with TAVR showed worse 1-year outcomes of CV death compared to TAVR without MS (adjusted HR 4.2, CI 2.4-7.38) and disabling CVA (adjusted HR 3.66, CI 1.36-9.91). TAVR with degenerative MS, when compared to TAVR with rheumatic MS, was associated with worse 1-year outcomes, with an adjusted HR 3.41, CI 1.20-5.81 for CV death and an adjusted HR 2.73 with a CI 1.15-6.49 for stroke. The etiology of this difference between the short-term and long-term prognosis of rheumatic and degenerative MS was not clear to the authors. However, they surmised that it could be due to a higher proportion of severe grades of MS in the rheumatic MS group [48]. There are case reports of TAVR in combination with either percutaneous mitral commissurotomy or transcatheter mitral valve implantation for combined rheumatic mitral stenosis and aortic stenosis [64-66].

More patients with RHD need to be included in prospective clinical trials of TAVR in the future, to help predict the outcomes and to determine the optimal options for treatment of TAVR with associated rheumatic MS.

### 3.4 The Effect of TAVR on MV Geometry

A few studies have investigated the changes in the geometry of the mitral valve after TAVR. Shibayama *et al* [67] studied 90 patients who had undergone TAVR with the Edwards SAPIEN valve for AS. They observed no significant difference in the geometry of the mitral annulus, either pre- or post-TAVR, with the TAVR seeming to improve mitral valve tethering. Another study by Vergnat *et al*. [68] included 20 patients and assessed the impact of SAVR and TAVR on mitral annulus geometry using 3D echocardiography. Amongst them, 10 patients who underwent TAVR had no change in the mitral annular geometry. However, the SAVR patients had reduced mitral valve transverse area, mitral annular height, and mitral annular area, respectively. Similarly, a study by Tsang *et al* [69] observed pre-TAVR and post-TAVR 3D echo findings in 33 subjects with severe AS who had undergone TAVR with a SAPIEN valve (Edwards Lifesciences, Irvine, CA) or a CoreValve (Medtronic, Minneapolis, MN). They noted that after TAVR, the mitral valve annulus was less spherical, suggesting that the prosthetic aortic ring was compressing the aortic mitral septum.

More studies and research are required to determine the geometric and hemodynamic effects of TAVR on the mitral valve annulus, especially in patients with concurrent MS [70, 71].

## 4 DISCUSSION

Atherosclerotic, degenerative, and aging processes predispose individuals to mitral and aortic valve stenosis. In the pre-TAVR era, surgery was the ideal intervention for patients with moderate to severe MS undergoing surgery for AS intervention. However, frailty, advanced age, and comorbid conditions render certain patients unsuitable for such invasive procedures. Transcatheter interventions have risen as less invasive options [72-74]. Among the aging and frail populations, TAVR is considered a more feasible treatment option for the correction of severe symptomatic AS. Given the increasing rate of TAVR procedures, meticulous patient selection is essential to maximize the advantages and mitigate the risks of this intervention. Multivalvular disease has an association with increased mortality and morbidity when compared to isolated valve disease. The diagnosis and classification of multivalvular cardiac disease still remains poorly defined, as it includes a wide spectrum of valve abnormalities with varied degrees of stenosis and regurgitant valvular pathologies. The differences in diagnostic criteria and quantification of severity add to the complexity of designing and comparing the results of various clinical studies [75-77].

The resulting hemodynamic changes complicate the echocardiographic evaluation of valve disease severity. Echocardiographic assessment of mitral stenosis severity, as measured by transmitral gradients and valve area, may be underestimated in patients with severe aortic stenosis, as the pressure half-time can overestimate the mitral valve area due to the presence of left ventricular diastolic dysfunction. The continuity equation could result in an underestimation of the mitral valve area due to its dependence on flow. Estimation of MVA by planimetry is also unreliable in patients with mitral annular calcification. Lastly, individuals who may appear to have concomitant MS and severe AS may present with “pseudo-severe” MS, which improves after TAVR for AS [57].

The procedure of choice for combined degenerative aortic and mitral stenotic disease is considered to be double valve surgery, but it is associated with poorer outcomes when compared to isolated valve surgical correction. Furthermore, in subjects with non-severe MS, surgical correction of the mitral valve may not be indicated, as patients may be managed medically instead. Therefore, professional societies and guideline groups have not been able to give recommendations regarding the management of multivalvular disease beyond a consensus level, that is, level C [44]. Recent trials have demonstrated that in subjects with TAVR, severe mitral stenosis (MS) is associated with worse outcomes, while mild to moderate MS shows no significant difference compared with isolated severe AS. The question remains: is there equipoise then, since some data report no impact of MS on TAVR, while others negate these findings? These findings largely serve to generate hypotheses, and the purported effects of MS on TAVR could be further affected by the higher risk profile of the patient population, concomitant mitral regurgitation (MR), along with associated baseline confounders and comorbidities such as age and frailty. The short- and long-term outcomes reported by various studies seem to support the clinical decision to intervene with TAVR, as outcomes did not differ significantly among those with concomitant MS and those without [78-82]. For example, the outcome difference noted by the STS/ACC study was surmised to be related to baseline characteristics of the subjects rather than the underlying MS [29]. Rheumatic heart disease, as opposed to MS severity, was the associated factor in the study using STAVI registry data [48]. Comparable functional status and exercise tolerance levels were also reported in this patient cohort.

It is important that decisions regarding the treatment of severe MS be made on an individual, case-by-case basis. The introduction of dedicated transcatheter mitral valve implantation devices may hold the key to improving outcomes in patients with coexisting aortic and mitral valve stenoses. There are reports of adverse effects when intervening on MS simultaneously with AS, due to acute left ventricular (LV) failure from a rapid rise in preload on a hypertrophied LV, which could potentially lead to pulmonary edema. On the other hand, hemodynamic studies suggest that the decrease in LV pressure following TAVR may lead to a low cardiac output state, which could be exacerbated by concomitant severe MS [83, 84]. Therefore, anticipating this postoperative complication and implementing appropriate therapeutic strategies, including adequate volume resuscitation and support with inotropes, may improve postoperative survival outcomes for these patients. More research and studies are needed to help ascertain the optimal management of these patients.

## CONCLUSION

Currently, many studies seem to indicate poorer long-term and short-term outcomes with performing TAVR in subjects with severe MS; however, not all studies have shown statistical significance. Clinicians should apply clinical judgment when deciding whether to opt for TAVR-only intervention for patients with concomitant severe MS and should perform further testing to assess the severity of MS and associated hemodynamics. Consideration should be given to either surgical valve replacement for both mitral and aortic valves, or combined *vs* staged TAVR and transcatheter intervention of the severely stenosed mitral valve in appropriate patients. Further high-powered and quality research is needed to evaluate the objective effect of different grades of MS on TAVR outcomes. Thus, well-designed, multicenter, prospective clinical trials with stratification of MS severity in patients undergoing TAVR, including short- and long-term outcomes, are needed to determine the best treatment strategy for TAVR with concomitant MS.

## Figures and Tables

**Fig. (1):**
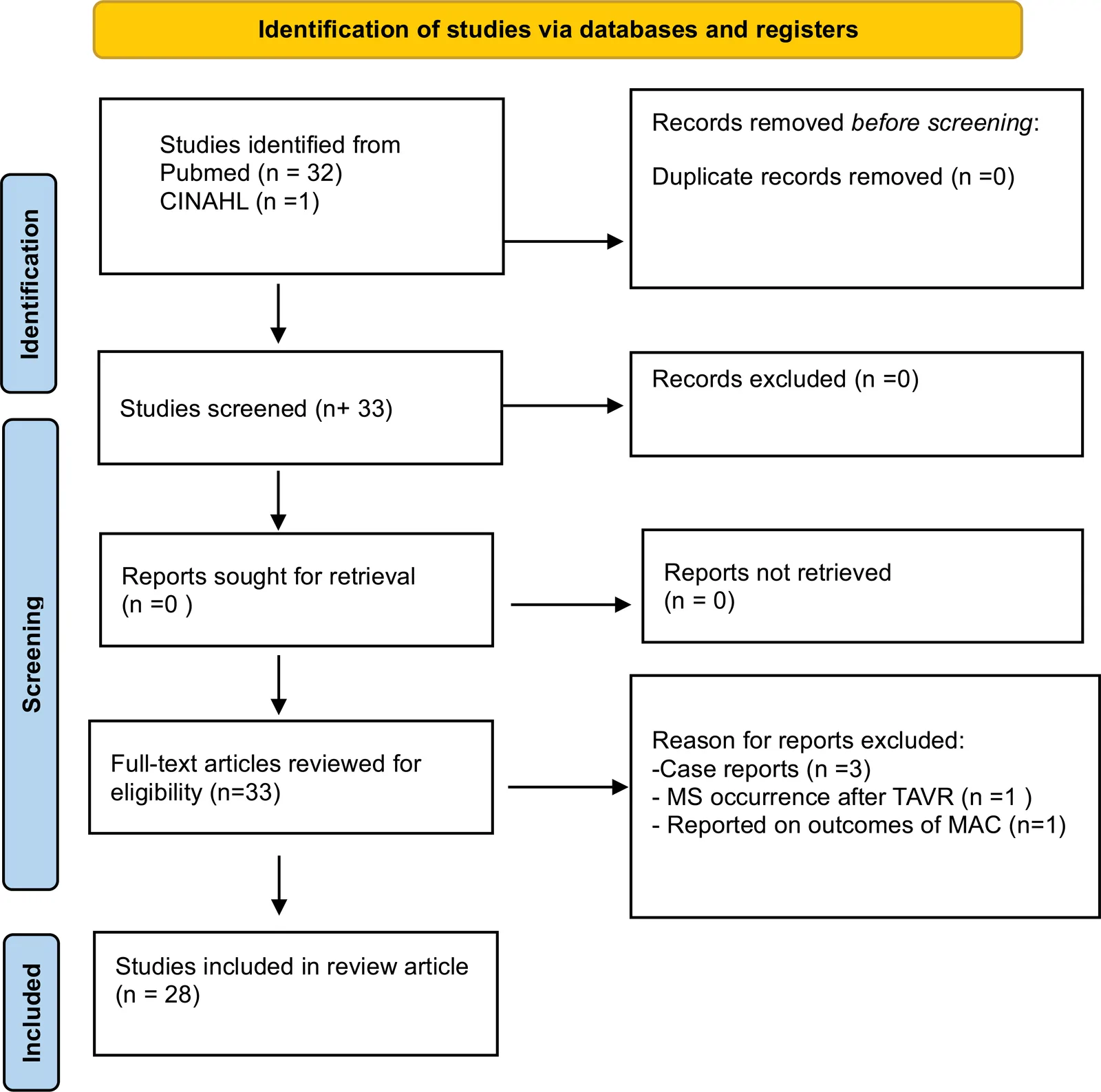
Literature review and the process of selection of studies. **Abbreviations:** MAC: Mitral annular calcification, MS: Mitral stenosis, TAVR: Transcatheter aortic valve replacement.

**Table 1:** Summary of characteristics and outcomes of studies of mitral valve stenosis in patients undergoing Transcatheter Aortic Valve replacement.

**Study, Year, Location**	**Study Type**	**Total no of TAVR Cases**	**Total no. of Patients with Concurrent MS (%)**	**Mean Age (Years)**	**Technique to Determine Valvular Heart Disease and Grading of Severity**	**Mean Follow-up**	**Outcomes**
Joseph *et al* [29], 2018 (USA)	Retrospective analysis of the TVT Registry 2011-2015	44,755	Severe MS: 1214 (2.7%) Non-severe MS 3987 (8.9%)	82.0± 8.5 81.6 ± 8.5	Echocardiographic and cardiac catheterization data severe MS ≤ 1.5 cm^2^ MVA Non-severe MS 1.51 - 4 cm^2^ MVA	1 year	Severe MS group, compared to the no MS group, had a higher risk for: • Primary outcome (composite of all-cause mortality, cerebrovascular accident, hospital admission for heart failure, and intervention for mitral valve disease at 12 months post TAVR) ( adjusted HR 1.2, *p*= 0.001), • 1-year mortality (adjusted HR 1.2, *p* = 0.046) • 1 year heart failure-related hospitalization (adjusted HR 1.3, *p* =0.009)
Asami *et al* [48], 2018 (Switzerland)	Retrospective analysis of the Swiss Transcatheter Aortic Valve Implantation Registry 2007-2015	971	MS: 176 (18.1%) Mild MS 148 (15.2%) Moderate/severe MS 28, (2.9 %) Degenerative MS=110 (62.5%) Rheumatic MS 66 (37.5%)	82.5 ± 5.8	TTE or TEE echocardiography. The severity of MS was graded using the mean MMG and MVA. MMG mild <5 mm Hg moderate 5–10 mm Hg severe >10 mm Hg valve area mild 1.5–2.5 cm^2^ moderate 1.0–1.5 cm^2^ severe <1.0 cm^2^	1 year	1-year outcomes: • CV death risk was increased in patients with MS* vs *no MS: 21.4%* vs *8.7% with a HR of 3.64, 95% confidence interval 2.38–5.56 • Disabling stroke was increased in patients with MS* vs *no MS: 7.1%* vs. *3.0%; adjusted HR 2.98, 95% confidence interval CI 1.46–6.09 • The difference in CV death and disabling stroke in MS* vs *non-MS groups emerged within 30 days of the procedure and was mainly driven by the outcomes of rheumatic MS
Sannino *et al*, [49], 2019 (USA)	Retrospective	901	Moderate MS 147(16.3%) Severe MS 17(1.9%) MVA ≤ 1.5 cm^2^: 123 MVA >1.5cm^2^: 767 (890 patients with complete MVA and f/u data	81.3	TTE The grading of the severity of MS was determined using MMG: MMG <5 mm Hg; MMG ≥5, to <10 mm Hg MMG ≥10 mm Hg. Also grouped by MVA <= 1.5 cm^2^* vs* MVA >1.5cm^2^	40.8 months	Primary outcome: all-cause mortality at 5 years was worse in MMG ≥ 10 mm Hg; HR 2.91 (*p*=0.02) Other outcomes: No difference in 5-year mortality when stratified by MVA No significant differences in 30-day and 1-year all-cause mortality
Al-khadra *et al*, [58], 2019 (USA)	Retrospective (analysis of the US National Inpatient Sample database)	62,110	MS: 887 (1.43%).	79.1 ±9.87	ICD-9-CM codes 394.0 and 396.0.	In hospital	Subjects with concomitant MS* vs *no MS had higher in-hospital mortality (5.1%* vs *3.5% adjusted odds ratio 1.455; *p* = 0.021), and more major adverse cardiac events (9.0%* vs *7.1% aOR 1.297; *p* = 0.040)
Fischer *et al *[50], 2019 (Canada & France)	Prospective	2,133	157 (7.4%) moderate to severe MS 22 pts (14% of patients with MS) had a MMG ≥10 mm HG Degenerative MS 88%	80 ± 9	Transthoracic echocardiography Moderate to severe MS was defined as a mean transmitral gradient ≥ 5 mm Hg in the absence of severe MR	3±2 years	Mortality at 30 days (3.8%* vs *5.5%), 3 ± 2 years ( 35%* vs *36.2%), or CHF re-hospitalization were similar in moderate to severe MS* vs *no MS groups. Pts with MS with a MMG ≥10 mm Hg had similar short and long-term outcomes compared to the rest of the study cohort.
Schaefer *et al*, [51], 2019 (Germany)	Retrospective	2582 TAVR 1058 TAVR with MAC or MS	Any MS 707/2582 (27%) Mild MS (528/1058 (49.9%) Moderate MS 168/1058 (15.9%) Severe MS 11/1058 (1%)	80	TTE MMG:(mild < 5 mm Hg, moderate 5-10 mm Hg, and severe > 10 mm Hg)	703.5 days	All-cause mortality at 30 days: Mild MS 5.9% Moderate MS 15% Severe MS 72.7% Moderate to severe MS (OR 7.75 p *p*<0.001) was an independent risk factor of 30-day survival post TAVR No difference in outcomes between the MAC-no MS and mild MS groups
Okai *et al*, [56], 2020 (Japan)	Prospective	156	MS 15 (9.6%)	83 (median)	TTE MS defined as mean MMA ≥5 mm Hg	72 hours- 2 years	Primary endpoint: new onset of acute decompensated heart failure within 72 hours post-TAVR Multivariable logistic regression analysis demonstrated that MS was associated with a higher incidence of acute decompensated heart failure 72 hours post TAVR ( adjusted OR 14.227, *p*=0.002) 2-year mortality was higher in patients with MS* vs *no MS. (Log rank *p*=0.011)
Mesnier *et al, *[55], 2021 (France)	Prospective	1177	MAC with MS 85 (7.2%)	80.8	TTE MS defined as MMG ≥5 mm Hg	1 year	MAC-MS but not MAC without MS was an independent predictor in multivariate analysis for all-cause mortality at 1-year post-TAVR. (adjusted HR 1.74, *p* =0.02) All-cause death at 30 days was higher in the MAC-MS group* vs *the no MAC group (adjusted HR 2.3, *p*=0.03) All-cause death at 1 year was higher in the MAC-MS group* vs *the no MAC group (adjusted HR 1.73, *p*=0.04) MS, regardless of MAC, was associated with a higher rate of all-cause death 1 year post-TAVR (adjusted HR 1.76, *p*=0.01)
Kurpad *et al, *[59] 2023 (USA)	Retrospective (analysis of US National Inpatient Sample database 2015-2020)	303,250	MS 2615 (0.86%)	78.17y ± 0.49	ICD-10 codes	In-hospital	In-hospital outcomes: Subjects with MS compared to subjects with no MS had a higher incidence of acute respiratory failure (8.8%* vs *4.89%, *p* = 0.001), complete heart block (13.54%* vs *9.36%, *p* = 0.01), and permanent pacemaker placement (8.03%* vs *6.03%, *p* = 0.05) A longer length of hospital stay (5.13* vs *3.85 days, *p* = 0.002) The in-hospital mortality was greater in the MS group* vs *no MS; but not statistically significant (1.32%* vs *1.53%, *p* = 0.679)
Ali *et al* 2024 [53] (USA)	Retrospective (analysis of The Nationwide Readmission Database 2016-2019)	217,147	MS 2140 (0.98 %)	78.6 y	ICD-10-CM codes	In hospital	Primary outcome: 30-day all-cause readmission rate TAVI with MS* vs *no MS had a higher rate of 30-day readmission (15.8%* vs *12.3%, aOR 1.22, CI: 1.03-1.45, *p* < 0.01) TAVI patients with MS* vs *no MS had longer lengths of hospital stay (5.7* vs. *4.3 days) In-hospital mortality was higher in the MS group, but there was no significant difference (2.1%* vs. *1.5%)

**Abbreviations:** MS, mitral stenosis, TTE: transthoracic echocardiogram, TEE: transesophageal echocardiogram, HR Hazard ratio, aOR adjusted odds ratio, ICD-9-CM, International Classification of Disease, Ninth Edition, Clinical Modification; MAC, mitral annulus calcification; MMG, mean mitral gradient; MVA, mitral valve area; AHA, American Heart Association; ACC, American College of Cardiology, TVT Transcatheter Valve Therapies Registry of the Society of Thoracic Surgeons /American College of Cardiology.

## LIST OF ABBREVIATIONS

AS: Aortic Stenosis
ADHF: Acute Decompensated Heart Failure
AVR: Aortic Valve Replacement
CI: Confidence Interval
CV: Cardiovascular
CVA: Cerebrovascular Accident
HR: Hazard Ratio
LV: Left Ventricle
LVOT: Left Ventricle Outflow Tract
MAC: Mitral Annular Calcification
MS: Mitral Stenosis
MAVPG: Mean Aortic Valve Pressure Gradient
MMG: Mean Mitral Gradient
MVA: Mitral Valve Area
RHD: Rheumatic Heart Disease
SAVR: Surgical Aortic Valve Replacement
TAVR: Transcatheter Aortic Valve Replacement

## STANDARDS OF REPORTING

PRISMA guidelines and methodology were followed.
